# Germline mutations in BRCA1 and BRCA2 among Brazilian women with ovarian cancer treated in the Public Health System

**DOI:** 10.1186/s12885-024-12246-1

**Published:** 2024-04-19

**Authors:** Caroline de Oliveira Ferreira, Vandré Cabral Gomes Carneiro, Carolline Araujo Mariz

**Affiliations:** 1 Hospital de Câncer de Pernambuco, Recife, Pernambuco Brazil; 2 Instituto de Medicina Integral Professor Fernando Figueira, Recife, Pernambuco Brazil; 3 Instituto Aggeu Magalhães, FIOCRUZ, Recife, Pernambuco Brazil; 4 Faculdade de Medicina de Olinda, Olinda, Pernambuco Brazil

**Keywords:** Brazil, BRCA1, BRCA2, Germline mutations, Ovary cancer

## Abstract

**Background:**

Germline mutations in BRCA1 and BRCA2 genes are among the main causes of hereditary ovarian cancer. Identifying these mutations may reduce cancer risk, facilitate early detection, and enable personalized treatment. However, genetic testing is limited in the Brazilian Public Health System, and data regarding germline mutations in many regions are scarce. Therefore, the study aimed to investigate the prevalence of germline mutations in BRCA1 and BRCA2 in women with ovarian cancer treated in the Public Health System in Pernambuco, Brazil.

**Methods:**

A cross-sectional study was conducted in the Hereditary Cancer Program from two reference oncological centers in Pernambuco. Women (*n* = 45) with high-grade serous ovarian cancer underwent genetic counseling and DNA sequencing for BRCA1 and BRCA2 genes.

**Results:**

The prevalence of deleterious mutations in the BRCA1 and BRCA2 genes was 33%. Of the 15 germline mutations found, 13 were in BRCA1 and 2 in BRCA2; two mutations of unknown clinical significance were also found in BRCA2. Mutations c.5266dupC and c.2215 A > T were the most frequent; each was mutation observed in three patients. Additionally, the mutations c.7645dupT and c.921dupT were reported for the first time.

**Conclusion:**

One in three women showed a pathogenic mutation, demonstrating a significant prevalence of germline mutations in this sample. Additionally, the small sample revealed an interesting number of mutations, indicating the need to explore more regions of the country.

## Background

 About 25% of ovarian cancers are hereditary [1], and around 65–85% of these cases are linked to germline mutations in BRCA genes [2]. These genes are tumor suppressors that maintain genome integrity, repair DNA, control the cell cycle, and regulate crucial steps of cellular division. Consequently, the loss of function of any BRCA protein increases genomic instability [3].

Germline mutations in BRCA1 and BRCA2 genes define the hereditary breast and ovarian cancer syndrome (HBOC), which presents an autosomal dominant inheritance pattern. This mutation is associated with a higher risk of breast and ovarian cancer (including fallopian tube and primary peritoneal cancer) and, to a lesser extent, prostate, pancreatic, and melanoma cancer. The latter is more observed in individuals with a BRCA2 mutation [4].

Individuals with a germline mutation in BRCA1 exhibit a chance of 48.3% for ovarian cancer by 70 years old (95% confidence interval [CI] = 38.8–57.9%) and of 20% (95% CI = 13.3–29.0%) when the mutation is in BRCA2 [5]. These estimates align with the Brazilian Society of Surgical Oncology and the National Comprehensive Cancer Network recommendations, which suggest genetic counseling and evaluation of BRCA1 and BRCA2 for all women diagnosed with epithelial ovarian cancer (including fallopian tube and peritoneal cancer), regardless of age [6, 7].

Identifying a germline mutation in BRCA1 and BRCA2 favors personalized treatments (e.g., poly ADP-ribose polymerase inhibitors), preventive strategies (e.g., genetic counseling, specific screening, and risk-reducing surgeries [e.g., salpingo-oophorectomy or mastectomy]), and a better cost-effective relationship for the health system [8–10].

Therefore, this study aimed to investigate the prevalence of germline mutations in BRCA1 and BRCA2 genes among women with high-grade serous ovarian cancer (HGSOC) treated in the Public Health System in Recife, Pernambuco.

## Methods

A cross-sectional study was conducted with patients of the Hereditary Cancer Program Hospital de Câncer de Pernambuco and Instituto de Medicina Integral Prof. Fernando Figueira in Recife. Both hospitals are referral centers for oncological treatment within the Public Health System from Pernambuco and other states in the Northeast region.

The study included women aged ≥ 18 years who had been diagnosed with HGSOC. They received genetic counseling before and after genetic testing, regardless of their family history. Germline mutations in BRCA1 (NM_007294) and BRCA2 (NM_000059) were assessed using DNA sequencing (next-generation sequencing); copy number variation was not assessed. Clinical data included age at diagnosis, family history of cancer, and location of origin.

Genetic analysis was performed via blood collection, and mutation status (i.e., frameshift, nonsense, missense, splice) was conducted using the ClinVar database (https://www.ncbi.nlm.nih.gov/clinvar/). Information regarding the identified mutations was obtained from genetic testing reports; however, those with negative results in sequencing were not searched for changes in copy number.

Data were collected from clinical records and analyzed regarding coherence and consistency using frequency tables. Statistical analysis assessed variable distribution using simple frequency with absolute and relative values. The research ethics committee of the Hospital de Câncer de Pernambuco (no. 82979518.2.0000.5205) and Instituto de Medicina Integral Prof. Fernando Figueira (no. 82979518.2.3001.5201) approved the study, and all women signed the informed consent form.

### Genetic analysis

Genomic DNA was extracted from peripheral blood by automation (QIASymphony platform, QIAGEN, Hilden, Germany) using the DNA Mini Kit extraction kit (QIAGEN). The DNA was sequenced by two or more complementary techniques to achieve 100% coverage of bases above 50x depth in all regions of interest. The quality and quantity of the extracted DNA were assessed by fluorometry (Qubit, Thermo Fisher, Waltham, Massachusetts). The genomic DNA was enzymatically fragmented and enriched, and fragments were barcoded via multiplexed PCR technology by QIAseq Targeted DNA Panels (CDHS 174272-2274 Qiagen). The first DNA library was designed using a multiplex polymerase chain reaction (PCR) kit (Qiagen). Subsequently, a second library was prepared using conventional PCR followed by Nextera (Illumina) to encompass regions not sufficiently covered by the first technique. Both libraries underwent second-generation sequencing (Illumina). A new Nextera library or Sanger sequencing was used if the 100% coverage was not achieved. Sequencing was performed on MiSeq or Next-Seq 550 instruments (Illumina, San Diego, CA) using MiSeq Reagent Kit v2 (300-cycles) or Next-Seq 550 High-throughput kit (300 cycles) with > 99% coverage at a minimum 50x depth.

The result covered 100% of bases with a depth above 50x in exons and a 10 bp region adjacent to the intronic region. Paired-end reads of 150 bp were aligned against the UCSC Genome Browser (hg19) and processed using a bioinformatics pipeline. Transcripts were numbered starting from the A base of the ATG initiation codon. Detected variants were classified as pathogenic, likely pathogenic, benign, likely benign, or uncertain significance [11]. When pathogenic mutations were identified, the findings were confirmed by Sanger sequencing using an ABI 3500 automated sequencer. Detection of c.156_157insAlu variant was performed according to Machado et al. (2007) [12]. Briefly, the PCR of BRCA2 exon three was performed, and the Alu insertion was detected by differential agarose gel electrophoresis.

## Results

Women with HGSOC (*n* = 45) underwent a genetic evaluation to investigate germline mutation. All were from the Northeastern region of Brazil: 42 from Pernambuco, 2 from Alagoas, and 1 from Paraíba. Diagnosis occurred between 19 and 69 years old (median age = 51); 31 reported a family history of cancer, mostly breast cancer.

Seventeen germline mutations were identified: 13 pathogenic variants or probably pathogenic variants in BRCA1 and 2 pathogenic variants or probably pathogenic variants in BRCA2; two variants of uncertain significance (VUS) were also identified in BRCA2. Among the women with mutations in BRCA1, the median age at diagnosis was 48 years old, and 12 had a family history of cancer. Additionally, three women with germline mutations in BRCA1 also had breast cancer (women 9, 10, and 13) (Table 1). Women 9 (41 years old) and 10 (61 years old) were diagnosed with triple-negative subtype concurrently with HGSOC. The Woman 13 (54 years old) was diagnosed with hormone receptor-positive breast cancer two years after HGSOC diagnosis.

**Table 1:** Mutations found in BRCA1 and BRCA2 genes in women with HGSOC

Women	Age	Gene	Location	Mutation	Type	Cases of familial cancer (age)
1	51	BRCA1	exon 13	c.4484G > T p.(Arg1495Met)	Missense	Breast (30; 59 and 63); lung (65); ovary (30); renal (60); stomach (89); and uterus (NI).
2	56	BRCA1	exon 13	c.4484G > T (p.R1495M)	Missense	NI
3	40	BRCA1	exon 10	c.2215 A > T p.(Lys739*)	Nonsense	Breast (30); breast (34); breast (35); uterus (57).
4	48	BRCA1	exon 10	c.2215 A > T p. (Lys739Ter)	Nonsense	NI
5	40	BRCA1	exon 10	c.2215 A > T; p.Lys739Ter (het)	Nonsense	Breast (40); breast (43).
6	42	BRCA1	exon 19	c.5266dupC (Gln1756Profs*74)	Frameshift	Breast (< 40); breast (> 50); breast (NI).
7	47	BRCA1	exon 19	c.5266dupC p.(Gln1756Profs)	Frameshift	No family history.
8	46	BRCA1	exon 20	c.5266dupc p.Gln1756Profs*74(het)	Frameshift	Intestine (50); ovary (48); prostate (60); CUP (60).
9	41	BRCA1	exon 11	c.1687 C > T p.GIn563Ter (het)	Nonsense	Ovary (46); uterus (35); cousin (NI).
10	61	BRCA1	exon 10	c.2761 C > T (p.Q921X)	Nonsense	Intestine (67); uterus (39).
11	66	BRCA1	exon 11	c.4165_4166delAG (p.S1389X)	Frameshift	Breast (35); breast (61).
12	48	BRCA1	intron 17	c.5074 + 2T > C (het)	Splice site	Breast (28); breast (44); intestine (> 50).
13	54	BRCA1	exon 10	c.921dupT (p.S309Ffs*6)	Frameshift	Breast (38); male breast (58).
14	57	BRCA2	exon 11	c.5611_5615del p.K1872Nfs*2	Frameshift	Ovary (NI); ovary (NI).
15	55	BRCA2	exon 16	c.7645dupT (het)	NI	Breast (> 50); prostate (30); thyroid (30).
VUS						
16	19	BRCA2	exon 11	c.5612G > A p.(Ser1871Asn)	Missense	No family history.
17	59	BRCA2	exon 16	c.7712 A > G; p.Glu2571Gly (het)	Missense	Lung (55); uterus (57); thyroid (59).

*Abbreviation*: *NI* Not informed, *CUP* Cancer of unknown primary, *VUS* Variants of uncertain significance

The variants identified, family history of cancer, and age are detailed in Table 1. Ten different deleterious germline mutations were detected. Mutations c.2215 A > T and c.5266dupC were the most frequent and presented in three women each, the latter described as the founding mutation of the Ashkenazi Jewish population; however, none of the women were Jewish. The c.2215 A > T mutation changes the lysine for a stop codon, resulting in a truncated protein. The c.5266dupC mutation involves duplication of a base, causing a loss of the reading frame at codon 1756 and a premature stop codon at the 74th subsequent amino acid.

The germline mutations c.921dupT (BRCA1) and c.7645dupT (BRCA2) have not been described in clinical or population frequency databases. The mutation c.921dupT involves a base duplication leading to a loss of the reading frame. Then, a serine is replaced by a phenylalanine at codon 309, resulting in a stop codon at the sixth subsequent amino acid. On the other hand, the mutation c.5074 + 2T > C was the only one occurring in an intron (or exon) junction region, potentially interfering with the splicing process.

All detected mutations were heterozygous. Regarding the type, 29.0% were nonsense, 23.5% missense, 35.0% frameshift, 6.0% splice sites, and 6.0% were not described in ClinVar.

## Discussion

The prevalence of germline mutation in the BRCA1 and BRCA2 genes was 33.3% (i.e., One in three women with HGSOC), characterizing HBOC. Some studies conducted in São Paulo reported a prevalence of 19.0% (*n* = 100), 20.8% (*n* = 158), and 30.0% (*n* = 50, ovarian cancer) [13–15]. Despite being a vast country with a possible wide range of data among regions, access to genetic evaluation is limited and unavailable in the Brazilian Public Health System. Consequently, the scarcity of studies hinders these comparisons.

Internationally, the prevalence of HBOC is lower: China (21.8%), Canada (13.3%), Colombia (15.6%), and Germany (20.8%) [16–19]. A study in Central West Florida revealed a prevalence of 15.3% of mutations in the BRCA1 and BRCA2 genes [20]. Another study from the United Kingdom and the Mayo Clinic in the USA found a combined prevalence in both genes of 11.0% in 2,222 women with HGSOC; the mutations were more common in this histological subtype [21]. Similar results were observed in Brazil, in which 24.5% of women with HGSOC presented a germline or likely germline mutation compared with 13.0% for other histological subtypes [14]. These findings support this study, which may have shown a higher frequency of germline mutations in a small sample because of the selection of this histological subtype.

The median age at ovarian cancer diagnosis was 51 years old for all women and 48 years old for women with germline mutations. Two studies in São Paulo showed higher median ages of 55 and 54.7 years old, respectively [13, 14]. Similarly, studies conducted in Belo Horizonte and the USA found a median age of 58.7 years old [22] and 56.6 years old [20], respectively. These differences might be related to the sample size and characteristics of each region, which could be further explored in larger samples.

In Latin America, founder mutations have been identified in Mexico (BRCA exon 9–12 deletion), Colombia (BRCA1 3450del4, A1708E, and BRCA2 3034del4), Latinos residing in Southern California (BRCA1 185delAG, IVS5 + 1G > A, S955x, and R1443x) Argentina (BRCA1 c.5266dupC), and Brazil (BRCA1 c.5266dupC and BRCA2 c.156_157insAlu) [23]. Among the founder mutations in Brazil, the BRCA1 c.5266dupC was the only one detected in our study in three individuals and is one of the most frequent BRCA1 and BRCA2 mutations [13–15, 24]. Similarly, a study assessed the spectrum of variants in 29,700 families worldwide found that 5266dupC is the most frequent in many European countries, such as France, Hungary, Russia, Poland, Italy, Latvia, Greece, Germany, and the Czech Republic [25]. The BRCA1 c.5266dupC mutation was initially described as a founder mutation in the Ashkenazi Jewish population. However, the high frequency in Europe raised questions about its origin [26]. Hamel et al. (2011) [26] genotyped 245 families from Brazil and Europe, demonstrating that individuals with the mutation shared a common haplotype from a single founder individual. Therefore, the mutation probably originated about 1,800 years ago in Scandinavia (Northern Russia) and spread to Europe. Thus, the mutation was introduced into the Ashkenazi Jewish population in Poland about 400 to 500 years ago.

A study conducted in Salvador sequenced the BRCA1 gene and identified two clinically relevant mutations: 3450del4 (also prevalent in Colombia) and p.R71G [27, 28]. Additionally, in Belo Horizonte, several mutations in BRCA1 (c.68_69delAG, c.5266dupC, c.181T > G, c.4034delA, c.5123 C > A) and BRCA2 (c.5946delT, c.8537_8538delAG, 4936_4939delGAAA) were assessed in women with ovarian cancer; none of the germline mutations were observed [22]. Therefore, the mutation c.5266dupC, the most common in Brazil, was not found in these studies. The analysis highlights the importance of genetic assessments in more patients. It also indicates that the search for specific mutations may not be suitable due to the substantial ethnic diversity of Brazil. Still, this search is an option in areas with limited resources,

The c.2215 A > T mutation found in a study conducted in São Paulo changes a lysine by a stop codon, producing a truncated protein [13]. The c.4484G > T mutation, the third most frequent, had previously been reported in Brazil [13, 14, 29] and was described as one of the most prevalent among African Americans [25]. In addition, the c.5611_5615del mutation was reported in a study with African Americans in the USA (in an individual without cancer). However, this mutation was not found in other Brazilian or international studies with a large sample [30].

The mutations c.4165_4166delAG, c.5074 + 2T > C, and c.1687 C > were frequently observed in other national studies [11, 13, 25], and the c.1687 C > T mutation was described as one of the five most common in Austria, Czech Republic, Finland, Germany, Italy, Lithuania, Sweden, and Brazil [23]. On the other hand, the c.2761 C > T mutation had not yet been described in Brazil but was reported in the USA and United Kingdom [21].

The mutations c.5612G > A and c.7712 A > G in BRCA2 (classified as VUS) had conflicting interpretations of pathogenicity in ClinVar; both showed assessments of uncertain significance and are possibly benign. Additionally, the mutations c.7645dupT in BRCA2 and c.921dupT in BRCA1 have yet to be reported in Brazilian studies or public databases. Thus, the results of this study are novel.

The results demonstrate a significant variety of germline mutations, confirming the heterogeneity within the sample and highlighting the need for more specific data from each region. Few studies in Brazil have assessed the genetic mutation profile in hereditary cancer, hampering the evaluation of specific characteristics related to ovarian cancer with germline mutations in BRCA genes. Most of these studies are focused on breast cancer and are concentrated in the South and Southeastern regions, and this study was the first conducted in Pernambuco.

The present study had some limitations. The small sample size from a single Brazilian region limited the generalization of prevalence rates, and a control group was not included for further comparisons. Moreover, a functional analysis of the novel germline mutations was not performed. Thus, we aim to increase the sample size and cover other Brazilian regions in future studies to address these limitations.

Individuals with non-mucinous breast and ovarian cancer have a 5.4-fold increased chance of carrying a germline mutation [31]. Identifying founder mutations is a cost-effective analysis aiming to reduce cost and enable genetic testing of many individuals [22] that rely only on the Public Health System. Additionally, the current study highlights the significant diversity of ancestry in the Brazilian population. Therefore, significant differences were observed, mainly due to the extensive territorial area and historical miscegenation. A specific screening, as performed in the Ashkenazi Jewish population, is challenging due to the scarcity of studies in the Northeast and other regions of Brazil. Additionally, the results did not show a significant recurrence of information that could identify predictors of germline mutations, which may be related to the small sample size. Therefore, further Brazilian studies must identify the profile of deleterious germline mutations associated with HBOC.

## Conclusion

Germline mutations were prevalent among women with HGSOC treated in the Public Health System of Pernambuco. In addition, novel data were found, indicating interesting characteristics in this sample. Thus, the results demonstrate the importance of monitoring and evaluating hereditary cancer, improving survival rates with an early diagnosis of breast cancer, and reducing the incidence of ovarian cancer in high-risk populations.

## Data Availability

No datasets were generated or analysed during the current study.

## Abbreviations

CI: Confidence interval
HBOC: Hereditary breast and ovarian cancer syndrome
HGSOC: High-grade serous ovarian cancer
PCR: Polymerase chain reaction
VUS: Variants of uncertain significance
